# A formative evaluation to inform integration of psychiatric care with other gender-affirming care

**DOI:** 10.1186/s12875-024-02472-8

**Published:** 2024-07-04

**Authors:** Teddy G. Goetz, Courtney Benjamin Wolk

**Affiliations:** 1 Department of Psychiatry, Perelman School of Medicine, University of Pennsylvania, 3535 Market Street, Philadelphia, PA 19104 USA; 2 Leonard Davis Institute of Health Economics, University of Pennsylvania, Philadelphia, PA USA

**Keywords:** Transgender, Non-binary, Integrated care, Mental health, Psychiatry, Primary care, Formative evaluation, Gender-affirming

## Abstract

**Background:**

Transgender, non-binary, and/or gender expansive (TNG) individuals experience disproportionately high rates of mental illness and unique barriers to accessing psychiatric care. Integrating TNG-specific psychiatric care with other physical health services may improve engagement, but little published literature describes patient and clinician perspectives on such models of care. Here we present a formative evaluation aiming to inform future projects integrating psychiatric care with physical health care for TNG individuals.

**Methods:**

In this qualitative pre-implementation study, semi-structured interview guides were developed informed by the Consolidated Framework for Implementation Research to ensure uniform inclusion and sequencing of topics and allow for valid comparison across interviews. We elicited TNG patient (*n* = 11) and gender-affirming care clinician (*n* = 10) needs and preferences regarding integrating psychiatric care with other gender-affirming clinical services. We conducted a rapid analysis procedure, yielding a descriptive analysis for each participant group, identifying challenges of and opportunities in offering integrated gender-affirming psychiatric care.

**Results:**

Participants unanimously preferred integrating psychiatry within primary care instead of siloed service models. All participants preferred that patients have access to direct psychiatry appointments (rather than psychiatrist consultation with care team only) and all gender-affirming care clinicians wanted increased access to psychiatric consultations. The need for flexible, tailored care was emphasized. Facilitators identified included taking insurance, telehealth, clinician TNG-competence, and protecting time for clinicians to collaborate and obtain consultation.

**Conclusions:**

This health equity pre-implementation project engaged TNG patients and gender-affirming care clinicians to inform future research exploring integration of mental health care with primary care for the TNG community and suggests utility of such a model of care.

**Supplementary Information:**

The online version contains supplementary material available at 10.1186/s12875-024-02472-8.

## Background

Transgender, non-binary, and/or gender expansive (TNG) individuals experience both disproportionately high rates of mental illness [1] and unique barriers to accessing care stemming from structural discrimination and minority stress (e.g., lack of provider knowledge about TNG-specific health needs, lack of provider cultural competence, provider discrimination, finances, non-inclusive infrastructure, and the sociopolitical environment) [2–5].

Some access barriers could be alleviated by integrating the delivery of mental health care with other gender-affirming care (GAC). Existing recommendations for improving mental health care for lesbian, gay, bisexual, transgender, queer, intersex, asexual, and other (LGBTQIA +) individuals emphasize the need for integrated models of care [6–8]. Our own prior work echoes this. In a previous qualitative study with TNG adults, this participant group expressed an overwhelming desire for mental health care that is integrated with GAC [9]. There may also be a synergistic benefit to such integration because GAC alone has been robustly demonstrated to improve depression and reduce risk of suicidal ideation and suicidal behavior [10–12]. In non-TNG patient populations, integrating mental health care with physical health care—particularly primary care—has been shown to improve clinical outcomes [13–15]. Specifically, the benefits of integrated models of mental health care, including the well-established Collaborative Care Model, have been noted for systemically marginalized and excluded populations [16, 17]. The theoretical benefits of integrated models of mental health care for TNG patients specifically have been suggested [18–20] (e.g. support navigating the health system, greater trust with clinicians, welcoming care environments, shared electronic health records including information about name/gender/pronouns, targeted training for clinicians on culturally-responsive care, better clinician understanding of community mental health needs) and there are examples of such clinics currently in practice [21]. However, little published primary literature describes the precise needs and preferences of TNG patients and gender-affirming clinicians from such a care delivery model [22].

Formative evaluations rigorously assess factors that may impact implementation to inform and guide implementation planning efforts [23]. Developmental formative evaluations focus on understanding the failings of current practice, imagined barriers to and facilitators of adopting a specific innovation, and perceived acceptability and feasibility of that innovation [24]. Here we present a developmental formative evaluation based on qualitative interviews with patient and clinician participants to inform the development and implementation of a model for integrating mental health, including specifically psychopharmacological, and physical health services for TNG individuals.

## Methods

### Procedures

Research participants were recruited by circulating flyers on private Facebook groups for TNG individuals and/or clinicians working in TNG health and by word of mouth, leveraging personal and professional networks outside of our institution (e.g., emailing the recruitment flyer to contacts who subsequently circulated it to individuals within their networks who may be eligible). Flyers are available in Appendix 1. Author TGG conducted 60-min semi-structured interviews via password-protected video chat after confirming eligibility and obtaining informed consent. Interviews were recorded and participants received a $20 gift card.

### Participants

Inclusion criteria (per self-report) were as follows. Patients: 1) TNG, 2) 18 + years old, 3) has experienced mental health disorder(s), 4) has accessed psychopharmacological care during their lifetime, 5) has sought some form of GAC—e.g., menstrual suppression, gender-affirming hormone therapy (GAHT), gender-affirming surgery (GAS) during their lifetime, and 5) living in the United States (US). Clinicians: 1) provide GAC, 2) cared for TNG patients experiencing mental health disorders, and 3) living in the US. We chose to limit patient participants to those who had previously accessed psychiatric medication management care because we wanted to specifically seek input on integrated mental health service models from individuals who had personal experience interfacing with mental health services (including psychopharmacological services) on which to draw. We wanted to interview clinicians with extensive expertise with this population to understand how mental health care is traditionally integrated and how that could be improved for this population. We also specifically chose to interview clinicians who provided GAC, rather than mental health services, to supplement the results of previous existing work that has already been conducted with mental health providers [9].

We recruited to thematic saturation, interviewing 11 patients and 10 clinicians [25]. Per Flanagin and colleagues (2021), we elicited data to facilitate understanding of qualitative interview responses in the context of individual intersectional positionality and to support diverse recruitment [26]. Race and ethnicity are reported collectively without specifiers of “biracial,” “multiracial,” or “mixed.” We collected participant demographic information via a REDCap screening survey (see Table 1).

**Table 1:** Participant demographics

**Patients (*n*** **= 11)**	
Mean age (SD); range in years	29.7 (5.3); (23–38)
Gender (n)	
Non-binary^a^	7
Trans man/transmasculine	4
Race and ethnicity (n)	
Asian/Pacific Islander	2
Black	2
White	5
Multiple^b^	2
Chronically ill/disabled	10
Mean # (SD); range of Mental Health Diagnoses^c^	4 (1.3); (2–6)
Past-year medical care (n)	
Primary care	11
Obstetrics and gynecology	5
Plastic surgery	5
Urology	2
Other	3
Prior GAC (n)	
GAHT	
*Ongoing use*	9
*Prior use; chose to stop*	1
*Seeking*	1
Chest GAS	
*Had*	7
*Seeking*	3
Genital GAS	
* Had*	2
* Seeking*	2
Hysterectomy	
*Had*	2
Geographic setting (n)	
Urban	7
Rural	4
Northeast	6
Northwest	1
Midwest	1
Southwest	3
**Clinicians (*n*** **= 10)**	
Mean age (SD); range in years	41.6 (8.1); (28–54)
Gender (n)	
Non-binary^a^	5
Cis woman	3
Trans woman	1
Cis man	1
Race and ethnicity (n)	
White	10
Clinical training (n)	
Physician	5
*Family medicine*	*2*
*Internal medicine*	*1*
*Obstetrics and gynecology*	*1*
*Naturopathic*	*2*
Family nurse practitioner	3
Midwife	2
Physician’s assistant	1
Mean (SD); range years since completing clinical training	11.9 (8.5); (< 1–30)
Clinical services (n)	
Primary care	9
Adult care	10
Children and adolescent care	3
GAHT	9
Puberty blockers	3
Fertility services	2
GAS	1
Practice setting (n)	
Academic	5
Community	4
Outpatient clinic	10
Private practice	3
Geographic setting (n)	
Urban	8
Rural	4
Northeast	3
Northwest	2
Mid-Atlantic	2
Midwest	1
Southwest	2
Mean (SD); range TNG % of all patients	69.2 (39.4); (5–100)
Mean (SD); range % of TNG patients with known mental health diagnoses^d^	58.5 (26.8); (10–100)

*SD* Standard deviation, *GAC* Gender-affirming care, *GAHT* Gender-affirming hormone therapy, *GAS* Gender-affirming surgery, *TNG* Transgender, non-binary, and/or gender expansive

^a^Non-binary gender term here includes one or more of the following terms: non-binary, transgender, genderqueer, transmasculine, gender non-conforming, femme

^b^Ashkenazi Jewish and white: 1; Asian and Black: 1

^c^For the purposes of this paper, we will not be considering “gender dysphoria” to be a psychiatric diagnosis and any diagnoses described by participants as in dispute/conflicting are reported here exclusively as the diagnosis that is most recent and perceived to be most accurate by the participant, and not counted multiple times. All patients had at least 2 diagnoses. Psychiatric diagnoses were as follows, alphabetically (n): attention deficit hyperactivity disorder (7), autism spectrum disorder (5), bipolar disorder (3), cocaine use disorder—in remission (1), eating disorder (3), functional neurologic disorder (1), generalized anxiety disorder (9), major depressive disorder (6), obsessive compulsive disorder (1), opioid use disorder—in remission (1), oppositional defiant disorder (1, in childhood), post-traumatic stress disorder (5), schizoaffective disorder (1), and social anxiety disorder (1)

^d^Clinicians with more TNG patients reported higher rates of known psychiatric diagnoses (*r* = 0.81, *p* < 0.01)

### Measures

We used the Consolidated Framework for Implementation Research (CFIR) [27–30] to develop group-specific semi-structured interview guides to facilitate uniform inclusion and sequencing of topics and to allow for valid comparison across interviews. Interview guides are available in (Appendix 2). At the beginning of each interview, participants were provided with a definition of integrated psychiatric care (see Appendix 2).

### Data analysis

We utilized a rapid analysis approach [30, 31]. The interviewer took notes during interviews on a structured summary template. Notes were then consolidated into a matrix organized by theme. The product was descriptive analyses of specific barriers to and potential optimization strategies for providing integrated gender-affirming psychopharmacological and other mental health care.

## Results

### Qualitative themes

Qualitative themes were organized by CFIR domains [28, 29]: (1) Outer setting: the broader context in which the inner setting exists, including systems-level factors and the socio-political environment; (2) Inner Setting: where the intervention is implemented, in this case clinics providing integrated psychiatric services for TNG individuals; (3) Individuals: the roles and characteristics of those involved with implementing a particular innovation or practice; and, (4) Innovation: characteristics and perceptions of the innovation or practice. Illustrative participant quotations are presented in Table 2.

**Table 2:** Representative participant quotations

Theme	Subtheme	Quote
Outer Setting	*Local Conditions*	*Barriers:*
		"[My psychiatry experiences to date have ranged from] nightmarish to affirming… [I've been] used as a learning experience." -patient (P15)
		"I never had a clinician use my correct pronouns… I had to fire [mental health clinicians] for lack of shared values… [like] repeated deadnaming me as part of exposure.” -patient (P71)
		"It's overwhelming trying to figure out, where do I go?" -patient (P77)
		Referring to sociopolitical climate and anti-TNG violence: "I just don't see the need for having someone in an office. In fact I find it's unsafe. Because if I'm gonna say to someone I want you to come to my office to see me and I've got a sign outside that says I do gender-related care, I am now putting that person at risk of bomb threats and other craziness. So for those reasons I'm not bringing anyone back in the office ever again." -clinician (P64)
		"Availability is the #1 barrier to care… [along with] lack of provider education." -clinician (P104)
		"Our [local referral option] services are a nightmare… [only] one in 10 get connected to those services and fewer access those services and I have absolutely no control… no faith that they'll have competence in trans and non-binary care." -clinician (P116)
	*Local Attitudes*	*Barriers:*
		"[I'm always worried going into psychiatric care:] If I need to change medications or increase doses, are they going to postpone or cancel my surgeries?" -patient (P71)
		"I didn't bring up my depression with my [PCP] because I didn't know what would happen.” -patient (P77)
		"I won't go to a place if I think the cops will be called on me for any reason." -patient (P92)
	*Partnerships and Connections*	*Recommendations:*
		“Queer and trans folks lead complex lives… the more resources [you can connect people to] the better.” -patient (P14)
		"Surgery is a temporary part of life… Mental health is such an integral aspect of overall health and my primary care has the best picture of my overall health." -patient (P71)
		"For surgical programs specifically, there needs to be more of an education process surrounding that... managing expectations, fluctuations, knowing enough to hold space and ask questions at certain intervals" -patient (P102)
		“Our job is not just provide care to the patient in front of us but to educate the community to become more accepting” -clinician (P109)
	*Policies and Laws*	*Barriers:*
		Regarding the necessity of fighting for healthcare reform, “Everything’s a band-aid on a gaping wound.” -clinician (P62)
		"Mental health has declined among [TNG] kids since these bans [on gender-affirming care have been introduced.]" -clinician (P65)
	*Finances*	*Barriers:*
		"It's hard to carve out time for communication, and insurance doesn't pay" -patient (P71)
		"[The barriers are] space, time, and money" -clinician (P1)
		"There's going to be a struggle with payment.” -clinician (P62)
		*Recommendations:*
		"[We need to have a point person in the clinic who] has a 30,000-foot view of finances." -clinician (P56)
		"Having a gender-affirming care program in place would ensure that [this psychiatrist] immediately has a panel and can demonstrate their usefulness to the clinic.” -clinician (P116)
	*External Pressure*	*Facilitators:*
		"This field is very much like they wanna see the numbers. And they wanna see like the citation… that could be something that could sway whoever is making the decisions on where… things should go in the future." -patient (P77)
*Inner Setting*	*Structural Characteristics*	*Recommendations:*
		"I'm a primary care stan [avid fan]… If you put it in Ob/Gyn, people will assume it's for AFAB people only; if you put it in plastic surgery, people who don't want surgery will think it isn't for them. Primary care is for everybody." -patient (P14)
		"[Telehealth should be offered because it would] save a lot of commuting time… [and] I have to work with my feelings of trauma to be sharing physical space with someone [due to fears about physical safety and COVID]. I feel safer [in telehealth appointments] which helps me go deeper." -patient (P71)
		“I live in a more rural area. I like being able to access my [gender-affirming] psychiatrist but having to go to [a city] every month isn’t feasible… I like being able to check-in with my psychiatrist [via telehealth] without disrupting my whole day." -patient (P102)
		"Most gender care can be given via telehealth" -clinician (P109)
		"A lot of folks feel more intimacy when they see their provider in person, but there are a million reasons why particularly trans folks wouldn’t feel safe or comfortable getting there in person." -clinician (P116)
	*Relational Connections*	*Barriers:*
		"Part of why I haven't followed up with psych care after being referred is not
		knowing who I'm being referred to… My PCP I know has had training in trans health and is non-binary… I don't know that a [general] psychiatrist would." -patient (P92)
		*Facilitators:*
		"An embedded model is ideal… you can vet the providers… It allows for more coordination of care and consulting" -patient (P10)
		"Accessibility of care… there are so many mental hurdles to even being open to seeking help for mental health… Going to the same place is going to facilitate a lot of connections [to care]." -patient (P77)
		"Being able to give a meaningful positive endorsement of the psychiatrist [is a huge benefit.]" -clinician (P116)
	*Communications*	*Facilitators:*
		"the greatest strength... the reason why I looked for this [integrated] model is ease of communication between providers. All my case notes are in the same medical record. There's no 'you can't talk to this person because they didn't sign the right consent form...having just one less thing to check off when you're struggling in the midst of depression to... have to tell... the psychiatrist the same thing I told the PCP." -patient (P12)
		"I am very interested in a model in which, with my consent, my PCP and psychiatrist are communicating and I don't have to manage all of that… I would love if my psychiatry and primary care were coordinated… It's a lot of work to be my own case manager, in a sense." -patient (P71)
		"It would be ideal if [my clinicians] all communicated… Being able to trust that my PCP has been able to relay information and that my psychiatrist got it and read it" -patient (P92)
		"Being able to communicate directly with the psychiatrist [would enable me to] provide better care for the patient by not having crossed wires." -clinician (P1)
*Individuals*	*Innovation Deliverers*	*Facilitators:*
		“If you work with a marginalized group there needs to be a commitment to their liberation” -patient (P14)
		“I think trans people seeing trans providers makes a big difference…I’m always checking the experiences and identities of my providers [before scheduling appointments with them.]” -patient (P71)
		“I’ve had the luxury of having a trans woman as my psychiatrist… It helps the trust factor and they have a good grasp of everything that goes into my healthcare.” -patient (P102)
		"Somebody dedicated who has this specific training and I don't have to train about pronouns would be great." -clinician (P56)
		*Recommendations:*
		"Anxiety, depression, PTSD [post-traumatic stress disorder], eating disorders, autism, and ADHD [attention deficit hyperactivity disorder]… are extremely prevalent in this population.” -clinician (P65)
	*Implementation Team Members*	*Barriers:*
		"I don't know what to do for people [with poor mental health] who don't have work, don't have housing" -clinician (P65)
		*Facilitators:*
		“For the people who we manage internally for psychiatry and behavioral health, I have been able to provide a fair amount of [staff] training [so I] have reasonable faith that the folks who are being seen internally are having a not outrageously bad experience.” -clinician (P116)
*Innovation*	*Relative Advantages*	*Facilitators:*
		"I'm all for making something streamlined and not having to go to seven places to get what you'd need. I'd love that." -patient (P6)
		Integrated psychiatric care is "the best way to go. I don't like operating in silos… [It's] more effective and efficient for everybody." -patient (P10)
		"I worry about [privacy] less than I would in a non-embedded system because I got there looking for that…. I'm a fan of not having to repeat my story five times." -patient (P12)
		"Psychiatry doesn't happen in a bubble. My mental health is best when my autoimmune disease is well-managed, when I'm on a good dose of T… mental health is a part of physical health." -patient (P15)
		"[A TNG-specific integrated psychiatric clinic] sounds like something a lot of people would be interested in…. I know a lot of people who had a hard time finding a psychiatrist. If I'd gone to this clinic and had a good experience, that's absolutely something I would tell them about." -patient (P92)
		"It's great to have [integrated psychiatric care] available… once things are titrated, stabilized, they can come back to me and I can manage that." -clinician (P104)
		*Recommendations:*
		"[There's a] dire need for good therapy and med management [for TNG folks.]" -clinician (P68)
	*Complexity*	*Barriers:*
		“I don’t think OCD is a diagnosis PCPs are comfortable with so I’d be concerned if the model was only for a short-term with psychiatry” -patient (P71)
		"Scope: [PCPs usually] can't manage serious mental health conditions" -patient (P92)
		"[Having psychiatric care managed by primary care alone in the past] worked fine for me, but there was less opportunity to tweak things. I have a lot of other things: diabetes, hypertension, so there isn't time to talk about [mental health] in my primary care appointments, so I'd like [a psychiatrist] separate within the same clinic [on a long-term basis]." -patient (P102)
		"Psychiatry has a higher acuity of patients that they're willing to manage." -clinician (P116)
	*Design*	*Facilitators:*
		Having psychotherapy on the same team brings “so much more depth” to the whole clinical team’s understanding of the patient and their needs. -patient (P10)
		*Recommendations:*
		"[Working with TNG patients] in such a low resource setting, I want every [psych service] possible." -clinician (P116)
		"I love groups and they can be helpful… [but] Groups haven't been well attended [in our clinic]… I think they have to be offered by organizations [where people want to go for that]." -clinician (P116)

### Outer setting

#### Local conditions

All participants cited cost as a major deterrent to individual engagement with psychopharmacological care. Transportation was another common barrier (most patients, some clinicians), especially for those living in rural areas. Participants were concerned about the paucity of gender-affirming psychiatrists in general (a few patients, most clinicians), and specific difficulty finding psychiatrists who can address multiple marginalized identities in their local community (two patients, a few clinicians). To protect clinicians and patients from anti-TNG violence, recommendations included: using a vague clinic name (one clinician), avoiding press coverage (a few clinicians), and using telehealth (a few clinicians).

#### Local attitudes

Participants identified patient mistrust of medicine in general, often warranted by TNG individuals based on previous negative experiences, as a barrier (two patients, some clinicians).

#### Partnerships and connections

To build trust with TNG patients, most patient and clinician participants described the importance of engaging with local LGBTQIA + community services and peer support organizations.

#### Policies and laws

Two clinicians cited anti-TNG legislature restricting access to GAC as an additional barrier to accessing appropriate psychopharmacological care; one clinician noted that legislative restrictions on telehealth disproportionately impact TNG patients attempting to access care. One clinician requested access to a legal team to help protect the medical licenses of the clinicians providing GAC and psychopharmacological care to TNG individuals.

#### Finances

Funding for services and billing constraints in U.S. healthcare were major challenges (one patient, most clinicians); most clinicians were concerned that smaller or rural practices would not be able to financially support dedicated psychiatrist time. Participants valued accepting both public and private insurance.

#### External pressure

Participants deemed institutional/systems support as important for practice reasons (e.g., ensuring adequate space) and for staff/patient safety (e.g., security against threats of violence). A few patients wanted their care teams to advocate for GAS for patients with higher body mass indices.

### Inner setting

#### Structural characteristics

All participants wanted integration of psychopharmacological services with primary care that specifically offered GAC (e.g., GAHT) to facilitate cohesive, streamlined care. All participants also wanted direct psychopharmacology appointments, in which the patient has an appointment with a psychiatrist who is integrated in a clinic where they already receive care; all clinicians and one patient additionally wanted the option of psychiatric consultation, in which a psychiatrist consults directly with clinicians on the patient’s primary medical treatment. Participants preferred longer timeslots for psychiatry appointments to build trust (one patient, some clinicians) and clear referral criteria (a few clinicians). Participants specifically requested clinic protocols for: providing letters of support for GAS (some patients, most clinicians), caring for patients after discharge from inpatient psychiatric admission (a few clinicians), assuming care for transitional age TNG patients from pediatric clinics (one patient, two clinicians), information-sharing between clinicians (one patient), police involvement (one patient), and screening patients for eating disorders (a few patients, one clinician), post-operative mood disturbance after GAS (one patient, two clinicians), and post-partum mood disturbance (one clinician). Two clinicians expressed concern that clinician turnover in the clinic could hinder the ability to monitor mental health symptoms over time.

Telehealth services were broadly desired (telehealth only: most patients, some clinicians; both telehealth and in-person options offered: one patient, most clinicians; in-person only: a few patients, no clinicians) and said to reduce transportation barriers to accessing care (most patients, most clinicians). No participants raised concerns about technological or connectivity requirements for telehealth, and those in rural settings specifically preferred such technologies.

#### Relational connections

Most patients and all clinicians were particularly excited about being able to refer patients to a specific, known psychiatrist, and minimal wait time to establish psychiatric care.

#### Communications

Most patients and all clinicians prioritized effective communication between the primary medical team and mental health clinicians to improve management of interconnected concerns, lab monitoring, and testing. Warm handoffs were noted to humanize patients to new clinicians and improve quality of care. A few patients and all clinicians valued protected time for clinicians to communicate about patients, coordinate care, and discuss intervention implementation. To this end, some patients and most clinicians wanted complete visibility of psychiatrist notes in the electronic health record to primary care providers (PCPs), and two patients and two clinicians feared that insufficient PCP/psychiatrist communication could delay or impede care; conversely, some patients and one clinician wanted limited visibility of psychiatrist notes for patient privacy.

### Individuals

#### Innovation deliverers

Participants specified the importance of clinicians being TNG-competent and TNG-affirming (most patients, all clinicians), with a particular preference for openly-TNG clinicians (most patients, some clinicians). Some patients and one clinician specifically preferred the option of having a shared race psychiatrist; most patients and a few clinicians noted that having multiple psychiatrists on the care team is ideal, as one clinician is unlikely to be a perfect fit for all patients. Participants requested competence with a disability justice framework, race, and a range of internalizing and externalizing conditions.

Participants described a trusting relationship with the primary clinical team as an important facilitator. One patient and two clinicians preferred a clinical team that was engaged in political advocacy on behalf of TNG rights and GAC. Two clinicians wanted psychiatric collaborators to be engaged in lobbying for more meaningful reimbursement of integrated psychiatric care for TNG persons to support the financial feasibility of this model of care.

#### Implementation team members

Participants wanted front-desk staff at the clinic to receive adequate training on being gender-affirming with name and pronoun use (one patient, one clinician). Most patients and most clinicians valued having support staff available to assist with coordinating patient care and community referrals; two patients and a few clinicians requested access to a social work team to help with housing, employment, disability paperwork, and other social service needs.

### Innovation

#### Relative advantages

Universally participants were interested in integrated psychopharmacological care with primary care settings that offer GAC and voiced the perceived appropriateness and feasibility of such an intervention over traditional siloed care models. The two patients and three clinicians who had personal experience with integrated psychopharmacological care all stated that it was their favorite model of care. Participants identified this high need/low resource patient population as particularly well-suited for integrated care because of the high prevalence of psychiatric medication use (two patients, three clinicians). All patients and most clinicians expected that an integrated model of care would lower the burden on patients of finding and establishing care with a psychiatrist; additional benefits included: patient comfort with referral to a known entity (some patients, most clinicians), an integrated psychiatrist for this population understanding fluctuations in mental health around GAC (some patients, most clinicians), synergistic mental health improvements for those receiving both mental health care and GAC (some patients, most clinicians), protecting patients from non-affirming experiences (two patients, most clinicians), and patients not needing to tell the same story repeatedly (some patients, two clinicians).

Some patients and most clinicians imagined that transitioning stable patients back to primary team management alone would save patient time and effort and healthcare resources. Most clinicians expected collaborating with an integrated psychiatrist to have spillover effects by improving PCP comfort managing psychiatric medications. Two patients and clinicians specifically predicted that fewer patients would be lost to follow-up from integrated care. Participants also envisioned benefits to providing additional mental health and/or gender-affirming services that are not typically offered in primary care alone.

#### Complexity

Participants were concerned about barriers to transitioning stable patients to PCP management of psychopharmacology, including PCP discomfort with psychiatric medications (some clinicians, one patient), patient discomfort with PCPs managing psychiatric medications (two patients, one clinician), and insufficient time in PCP appointments to adequately address psychiatric needs (some clinicians, one patient); inability to transition stable patients to PCP management in turn could reduce availability for new patients to see psychiatry (most patients, two clinicians). Two patients worried that there could be an excessive patient volume for the psychiatric team (given high need and limited access in the community) to provide optimal care and follow-up, which could delay access to care.

One patient and a few clinicians noted that an integrated psychiatry model relies upon the mental health screening that identifies patients for referral. Participants did not deem time-limited care (e.g., fertility care, gender-affirming surgery) ideal for integrated psychopharmacological care (one patient, two clinicians). Two patients and clinicians voiced concern that integrating psychiatric medication management with procedural care (e.g., GAS, fertility care) posed the risk of patients withholding information about mental health symptoms to avoid being denied access to such procedures.

#### Design

There was robust interest in optional individual psychotherapy being offered by a licensed mental health professional working as a member of the same integrated care team (most patients, all clinicians), including longer-term psychotherapy (most patients, all clinicians). The two patients who preferred to access psychotherapy from a licensed mental health clinician working separately from their other medical care cited privacy concerns. There was mixed interest in group psychotherapy: two patients and a few clinicians wanted LGBTQIA + -specific psychotherapy groups (e.g., a dialectical behavioral therapy skills group); two clinicians worried about low enrollment based on their prior experiences. One clinician wanted psychotherapy groups for family members of TNG youth and separate psychotherapy groups for TNG youth. One patient and two clinicians wanted peer support groups that were not specifically psychotherapy. Two patients and clinicians wanted an LGBTQIA + -specific intensive outpatient program or partial hospitalization program to be offered.

Participants requested integrating additional services including obstetrics and gynecology care (a few patients, a few clinicians), care for TNG adolescents (two patients, a few clinicians), nutritionist (a few patients, one clinician), and other LGBTQIA + -targeted clinical services (one patient, some clinicians).

## Discussion

Patients and clinicians reported broad enthusiasm for integrated models of psychiatric care, which have the potential to improve care for TNG individuals and reduce barriers to finding and engaging with TNG-competent mental health clinicians. These findings are consistent with prior recommendations for exploring such models of care to meet the unique mental health needs of this population [6–8, 18–20]. Participants anticipated that integrated models of care could reduce experienced structural discrimination and minority stress, and were specifically interested in integration of psychiatric medication management with primary care settings that provide GAC (e.g., GAHT); family medicine may be a particularly good fit by providing care to TNG adolescents and adults and offering obstetric and gynecological services [32].

Participants universally desired psychiatrist medication management. Transitioning stable patients back to primary care alone would maintain availability of services to new patients, though participants voiced concern that PCPs might not be comfortable managing certain psychiatric conditions or medication regimens [33], underscoring the need for psychiatric appointments in addition to consultation. Participants also prioritized offering psychotherapy [3] and training all clinical team members in gender-affirming practices [3, 32, 34]. Telehealth options were cited as particularly desirable for systemically marginalized and excluded patient populations [35, 36], including TNG individuals [37, 38].

Study limitations include using an exclusively qualitative dataset, heterogenous participant exposure to integrated models of mental health care, and participant cohort diversity being restricted to those who responded to our call for participants—which notably did not include any trans women. The clinician cohort was limited to non-mental health clinicians. Future work is needed exploring these topics with systemically marginalized and excluded communities, particularly with Black and indigenous people of color clinicians, mental health clinicians, and trans women patient participants. The patient cohort was limited to participants who had previously interfaced with mental health care and cannot be generalized to all TNG individuals. Additionally, the interviews and coding were completed by one researcher to maintain consistency; however, we acknowledge that this procedure could introduce biases.

Building from our findings and the extant literature, we offer the following six recommendations for gender-affirming integrated mental health care:

- First, preference opportunities to integrate mental health services with settings like primary care/family medicine, where gynecological care and GAHT are offered and services are available for adolescents and adults. This has the advantage of leveraging patients’ longest-term healthcare relationship and most frequent point of contact, coordinating psychopharmacological management with GAHT and other medications, coordinating around reproductive needs, and meeting the needs of transitional age youth, which our clinician and patient participants overwhelmingly valued.
- We also recommend that, as possible, psychiatric medication management/consultation and psychotherapy services be offered in the same location. Our participants recognized the value of each of these services for this patient population generally, and the value of increasing care team communication and collaboration around psychopharmacology and psychotherapy specifically.
- Additionally, when possible, offering telehealth options may help increase access [39–41]. Telehealth services were widely identified by our patients as an opportunity to reduce transportation barriers to care, increase access for disabled patients and those in rural areas, and reduce scheduling constraints, which can be most impactful for low-income patients. Further, telehealth was noted to insulate patients and clinicians from anti-TNG violence.
- It will be critical for clinics to train care team members—including nursing, medical assistants, and front office staff—in gender-affirming communications. Gender-affirming interactions were broadly valued by participants, and the opportunity for training staff to provide respectful and affirming care was identified as a specific strength of providing population-specific clinical services for TNG individuals [42–44].
- Clinicians on the care team should have dedicated training in specific mental health considerations for TNG patients. Participants overwhelmingly cited the paucity of medical education on TNG mental health and highly valued the presence of clinicians with expertise in TNG mental health on such an integrated care team. Specific topics for clinician training could include: (a) Metabolic interactions between GAHT and psychiatric medications; (b) Perioperative management of psychiatric medications around GAS; (c) Influences of common physiological (e.g., post-operative depression, premenstrual exacerbations) and psychosocial (e.g., declined from desired GAC, anti-TNG legislation, impeded legal or social transition, non-affirming interactions) stressors; (d) Population-specific management of disorders not appropriately addressed by standard treatment paradigms (e.g., eating disorders); and (e) Basic understanding of GAS procedures and comfort providing letters in support of GAS, as required by surgeons and insurance companies [45].
- Finally, advocacy for public and private insurance coverage of integrated services is needed. Our participants told us that it was critical that integrated mental health services be covered by insurance. These findings constitute a starting point. We are currently conducting a pilot study informed by this formative evaluation and look forward to refining these recommendations based on that data.

## Conclusions

This formative evaluation offers an example of eliciting patient and clinician input to inform targeted health interventions for TNG communities and suggests utility of future research guiding models of mental health care integrated with gender-affirming primary care.

### Supplementary Information

Supplementary Material 1.

Supplementary Material 2.

## Data Availability

No datasets were generated or analysed during the current study.

## Abbreviations

CFIR: Consolidated Framework for Implementation Research
GAC: Gender-affirming care
GAHT: Gender-affirming hormone therapy
GAS: Gender-affirming surgery
LGBTQIA +: Lesbian, gay, bisexual, transgender, queer, intersex, asexual, and other
PCP: Primary care provider
TNG: Transgender, non-binary, and/or gender expansive
US: United States
