# Supplementary figures and images for: A formative evaluation to inform integration of psychiatric care with other gender-affirming care
Source: BMC Prim Care. 2024 Jul 4;25:239. doi: 10.1186/s12875-024-02472-8 (PMC11225323; doi:10.1186/s12875-024-02472-8)

Appendix 1: Recruitment Flyers


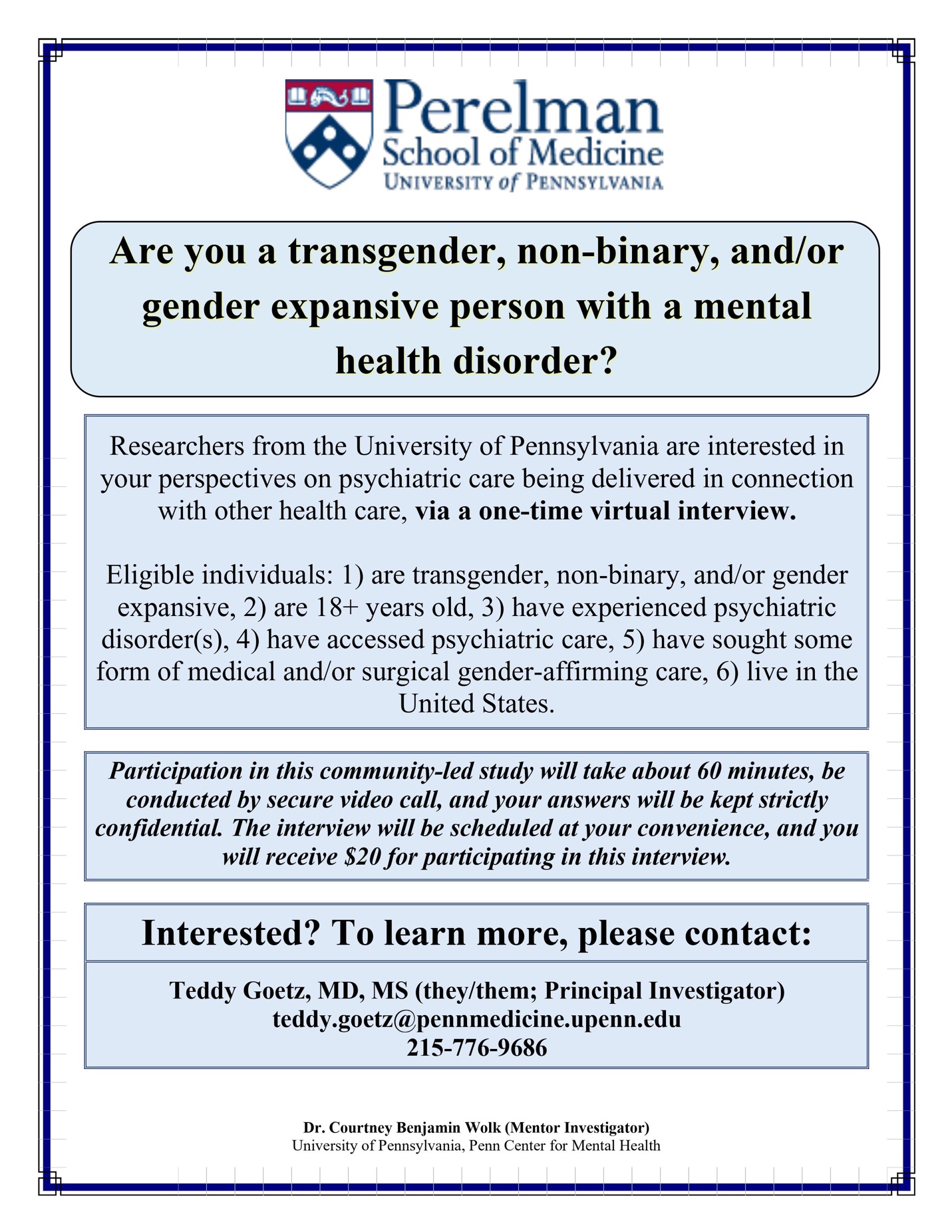


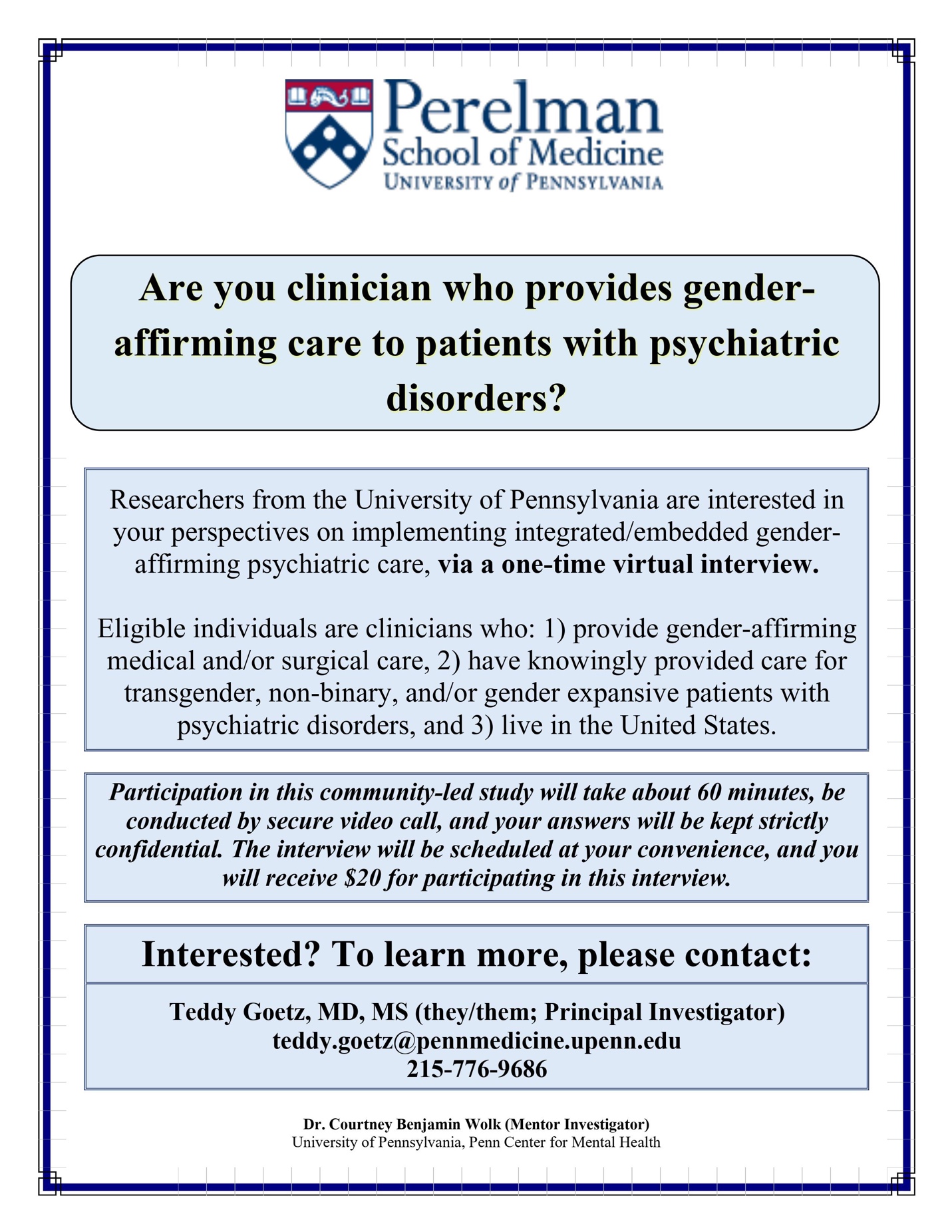

Supplement: Supplementary file 1 — Supplementary Material 1. [file 12875_2024_2472_MOESM1_ESM.docx]
