# Supplementary material for: A formative evaluation to inform integration of psychiatric care with other gender-affirming care
Source: BMC Prim Care. 2024 Jul 4;25:239. doi: 10.1186/s12875-024-02472-8 (PMC11225323; doi:10.1186/s12875-024-02472-8)
Supplement: Supplementary file 2 — Supplementary Material 2. [file 12875_2024_2472_MOESM2_ESM.docx]

Appendix 2: Interview guides

Patient group:

1) To frame our conversation today, would you please share which psychiatric diagnoses you have received during your life and which ones you currently hold?

2) To frame our conversation today, would you please share which forms of medical or surgical gender-affirming care you have received or are considering pursuing in the future?

3) What has your experience been like accessing psychiatric care in the past?

4) Psychiatric care can be delivered in different ways. Traditionally, psychiatrists have worked independently from any other doctors that someone might be seeing. For the purposes of this interview, we’ll talk about integrated models of care as signifying a psychiatrist works closely in collaboration with another one of the patient’s medical providers (e.g., primary care provider, gynecologist, surgeon) to help provide mental health care based out of that office (e.g., primary care, gynecology, surgery). In such a model, the psychiatrist and the other medical provider discuss any concerns they have about the patient and can coordinate ordering of any tests and medications. Sometimes the psychiatrist has appointments with the patient and sometimes they just talk to the other medical provider to discuss their specific concerns. Sometimes there is a psychotherapist who also works with the psychiatrist and other medical provider too, but not always. How interested would you be in receiving your psychiatry care as part of an integrated team like that?

5) What concerns would you have about integrating psychiatric care with other medical care? Are those concerns specific to this model of care or do they apply to any psychiatric care?

6) What strengths do you see in integrating psychiatric care with other medical care? Are those strengths specific to this model of care or do they apply to any psychiatric care?

7) What barriers do you imagine could prevent you from engaging in psychiatric care integrated with other medical care? Are those barriers specific to this model of care or do they apply to any psychiatric care?

8) What do you imagine could make it easier for you engaging in psychiatric care integrated with other medical care? Are those facilitators specific to this model of care or do they apply to any psychiatric care?

9) We’ve been talking about integrating psychiatric care with other medical care generally. When you imagine being a patient using this model of care, where would you want it to be integrated? Primary care? Gender-affirming surgery? Somewhere else? Why?

10) When you imagine being a patient using this model of care, how much interaction would you prefer to have with the psychiatrist? No face-to-face (your other medical provider talks to them only), one appointment, a few appointments, ongoing care? Why?

11) When you imagine being a patient using this model of care, would you want psychotherapy services to be offered within that integrated team as well? Why?

12) When you imagine being a patient using this model of care, what specific needs would you have from the clinical team delivering that care?

Clinician group:

1) To frame our conversation today, would you please share which gender-affirming clinical services you provide?

2) To frame our conversation today, what challenges have you faced in treating TNG patients with psychiatric disorders?

3) What has your experience been like collaborating with mental health clinicians in the past?

4) Psychiatric care can be delivered in different ways. Traditionally, psychiatrists have worked independently from any other doctors that someone might be seeing. For the purposes of this interview, we’ll talk about integrated models of care as signifying a psychiatrist works closely in collaboration with another one of the patient’s medical providers (e.g., primary care provider, gynecologist, surgeon) to help provide mental health care based out of that office (e.g., primary care, gynecology, surgery). In such a model, the psychiatrist and the other medical provider discuss any concerns they have about the patient and can coordinate ordering of any tests and medications. Sometimes the psychiatrist has appointments with the patient and sometimes they just talk to the other medical provider to discuss their specific concerns. Sometimes there is a psychotherapist who also works with the psychiatrist and other medical provider too, but not always. How interested would you be in working within an integrated model of care like that, in general and specifically when providing care for TNG individuals?

5) What concerns would you have about integrating psychiatric care with your clinical practice?

6) What strengths do you see in integrating psychiatric care with your clinical practice?

7) What barriers do you imagine could impede your integration of psychiatric care with your clinical practice?

8) What do you imagine could facilitate your integration of psychiatric care with your clinical practice?

9) What do you imagine are the pros and cons of integrating psychiatric care with your clinical settings vs. alternative clinical settings? Why?

10) When you imagine being a clinician working in this model of care, how much direct interaction would you prefer that your patients have with the psychiatrist? No face-to-face (consultation only), one appointment, a few appointments, ongoing care? Why?

11) When you imagine being a clinician working in this model of care, would you want psychotherapy services to be offered within the integrated team as well? Why?

12) When you imagine being a clinician working in this model of care, what specific needs and preferences would you have from the psychiatry clinicians collaborating with you?
